# The effect of activation of the sequential use of ethylenediaminetetraacetic acid and octenidine dihydrochloride on the release of TGF-β1 in regenerative endodontic treatments

**DOI:** 10.1007/s10266-026-01313-7

**Published:** 2026-01-18

**Authors:** Eda Doğuş, Emre Avcı, Maria Teresa Arias-Moliz, Sıla Nur Usta

**Affiliations:** 1 Department of Endodontics, Gulhane Faculty of Dentistry, University of Health Sciences, Ankara, Turkey; 2 Department of Biochemistry, Faculty of Pharmacy, University of Health Sciences, Ankara, Turkey; 3 Department of Microbiology, School of Dentistry, University of Granada, Granada, Spain

**Keywords:** Endodontics, Irrigation activation, Octenidine dihydrochloride, Regenerative endodontic treatment, Transforming growth factor

## Abstract

The aim of this study is to assess the effect of ultrasonic (PUI) and sonic (EDDY) activation of the sequential use of ethylenediaminetetraacetic acid (EDTA) and octenidine dihydrochloride (OCT) on the release of transforming growth factor beta-1 (TGF-β1) from dentin in regenerative endodontic treatments (RET). Seventy-two extracted were decoronated and prepared to an apical diameter of 1 mm to replicate an immature teeth. Each specimen was initially irrigated with 20 mL of 1.5% sodium hypochlorite according to the RET protocol. Samples were assigned to three main irrigant groups (*n* = 24): 17% EDTA, 0.1% OCT, and 17% EDTA followed by 0.1% OCT. Each main group was further divided into three subgroups (*n* = 8) based on activation technique—syringe-needle irrigation (SNI), PUI, and EDDY. Levels of TGF-β1 released on days 1 and 7 were quantified by ELISA, and smear layer removal was analyzed under scanning electron microscopy (SEM). Statistical significance was established at *p* < 0.05. SEM analysis revealed varying degrees of smear layer removal in all groups. The combination of EDTA and OCT resulted in significantly higher TGF-β1 release compared to the use of either solution alone in all activation groups (*p* < 0.05). TGF-β1 levels were significantly higher at day 7 than day 1 (*p* < 0.05). EDDY activation with the sequential use of EDTA and OCT yielded the highest TGF-β1 levels and the greatest percentage increase (*p* < 0.05). Biocompatible irrigation solutions and enhanced activation systems could improve the release of growth factors from dentin, thereby potentially contributing to the overall success of RETs.

## Introduction

Pulpal necrosis in immature teeth may arise due to bacterial invasion, traumatic injuries, or developmental anomalies, leading to the loss of odontoblastic viability and subsequent disruption of normal root development [1]. Teeth with incomplete root development are characterized by wide root canals, open apices, and thin dentinal walls, conditions that frequently compromise the effectiveness and prognosis of conventional endodontic treatment [2]. To address these limitations, regenerative endodontic treatments (RET) are defined as biologically based procedures aimed at restoring damaged structures—such as dentin, root tissues, and cells of the pulp-dentin complex—by promoting the regeneration of tissues that closely resemble the natural pulp-dentin complex [3].

In RETs, continued root maturation is achieved through the interaction of three key components, including stem cells, growth factors (GFs), and scaffolds integrated within a tissue-engineering paradigm [4]. Among them, GFs constitute a class of signaling molecules that play a crucial role in regulating key cellular processes, including migration, proliferation, and differentiation [5]. In this sense, dentin serves as a biologically active reservoir for GFs, which can be released through disinfection protocols during RETs [6]. Transforming growth factor beta-1 (TGF-β1), a key GF primarily localized within the peritubular dentin, plays a critical role by modulating immune responses, promoting chemotaxis, and regulating the proliferation and differentiation of stem cells, odontoblast formation, and dentin matrix secretion [7]. Since the presence of infected tissue in the root canal system exerts a limiting effect on the migration, adhesion, and proliferation capacity of stem cells, the successful realization of these biological processes is closely dependent on effective disinfection of the root canal system, highlighting the pivotal role of irrigation protocols in RETs [8].

Irrigation solutions play a dual role in RETs by exerting antimicrobial effects and facilitating the release of GFs from dentin into the surrounding microenvironment [9]. In alignment with this, a protocol involving initial irrigation with a low concentration (1.5%) sodium hypochlorite (NaOCl), followed by the application of 17% ethylenediaminetetraacetic acid (EDTA) to optimize regenerative outcomes was advocated [10, 11]. In this sense, EDTA is particularly indicated since it enhances the release of GFs from dentin by removing the smear layer, thereby exposing the underlying dentinal matrix [12]. However, concerns have been raised regarding its potential negative impact on cell behavior, such as impairing stem cell viability and migration, which may compromise the regenerative process [13]. Additionally, EDTA’s anticoagulant properties can hinder blood clot formation [14], and its interaction with NaOCl may reduce the latter’s antimicrobial efficacy [15]. Given these drawbacks, the search for alternative irrigation solutions instead of EDTA with more favorable biological and chemical profiles has gained increasing attention in RETs. One of these solutions, octenidine dihydrochloride (OCT), is a biocompatible antiseptic agent with strong antimicrobial activity against a broad range of microorganisms and low cytotoxicity [16, 17]. Its ability to support cell migration and reduce NaOCl-induced cytotoxicity suggests its potential as an alternative irrigation solution in RETs [18].

Given the minimal or absent mechanical instrumentation in RETs, widely used conventional syringe–needle irrigation (SNI) often proves inadequate for achieving thorough disinfection of the root canal system [8]. Therefore, sonic and ultrasonic activation systems are frequently employed to enhance the efficacy of irrigation since these techniques can increase the release of GFs by facilitating the removal of the smear layer and debris, as well as reducing the microbial load within the root canal system [19].

Although OCT has recently been proposed as a promising irrigant due to its potent antimicrobial efficacy and favorable biocompatibility [18], its capacity to facilitate the release of dentin-derived GFs remains largely unexplored. Given that EDTA is currently considered indispensable in RETs, complete replacement of EDTA with OCT may not be clinically feasible. Therefore, evaluating OCT not only as a standalone irrigant but also in sequential use with EDTA may provide a more clinically relevant approach. Such a strategy may allow preservation of EDTA-induced dentin demineralization while potentially mitigating its biological limitations through the adjunctive use of OCT. Moreover, the impact of irrigation activation systems on the bioactive potential of OCT has yet to be fully elucidated. Thus, this study aimed to assess the effect of sonic (EDDY) and ultrasonic (PUI) activation of EDTA, OCT, and sequential use of EDTA and OCT solutions on the release of TGF-β1 from dentin in RETs. The null hypothesis of the study was that there would be no difference between the solutions and activation systems used.

## Materials and methods

The study design was approved by the University of Health Sciences (no.: 2023–142). A power analysis was performed using G*Power v3.1 (Heinrich Heine, Universität Düsseldorf, Germany). Based on a similar study [20], with an effect size of 0.536, a significance level of 0.05, and a power of 85% the total required sample size was calculated to be 72.

### Sample preparation

This study was conducted using the materials and methods described by Usta et al. [21]. Seventy-two freshly extracted, single-rooted human teeth with straight canals and mature apices were collected from healthy (ASA I or II) individuals aged 18–50 and examined under a dental operating microscope to exclude any with cracks, root fractures, resorptive defects, or developmental anomalies. Residual periodontal tissues on the external root surfaces were gently cleaned using curettes. The specimens were then sterilized in an autoclave at 121 °C and 15 psi for 30 min. After sterilization, they were kept in a 0.1% thymol solution at 4 °C. All procedures commenced within one week after extraction to reduce the risk of storage-induced alterations.

The teeth were decoronated to obtain standardized root segments measuring 13 mm in length. To simulate an immature open-apex configuration and achieve the critical apical diameter of 1 mm, as recommended for RETs [22], the root canals were prepared using K-type stainless steel hand files up to size #100. During the instrumentation, canals were irrigated with distilled water between each file to ensure debris removal and maintain canal patency. The external surfaces of the teeth were coated with nail varnish and then placed in silicone-based impression material (Zetaplus L Intro Kit, Zhermack S.p.A., Italy) within sterile Eppendorf tubes to facilitate handling and simulate apical backpressure. Subsequently, the root canals were irrigated with 20 mL of 1.5% NaOCl and 20 mL of distilled water using a 27G needle, following standard protocols for RETs [10].

### Irrigation process

The root segments were randomly assigned into three main groups (*n* = 24 per group) according to the irrigation solution used: 17% EDTA (pH: 7), 0.1% OCT (pH: 6), and 17% EDTA followed by 0.1% OCT. Randomization was performed using randomizer.org. Each main group was further subdivided into three subgroups (*n* = 8 per group) based on the irrigation activation method, as described below:

*SNI*: Irrigation was performed using 27G disposable needles, positioned 1 mm short of the working length (WL) and moved in a gentle in-and-out motion to enhance solution distribution. A total of 5 mL of the assigned irrigant was delivered into each canal over a standardized duration of 20 s.

*PUI*: A #25 stainless steel ultrasonic file (Irri-Safe; Acteon, Mérignac, France) was connected to an ultrasonic device (Newtron B.LED XS; Satelec Acteon, Mérignac, France) and inserted to a position 2 mm short of the WL. For each sample, 5 mL of the designated irrigation solution was ultrasonically activated for 20 s.

*EDDY (VDW, Munich, Germany)*: A non-cutting polyamide tip (#25) was mounted on an air-driven sonic handpiece (Kavo Kerr, Detroit, USA) and placed 2 mm short of the WL. The assigned 5 mL of irrigation solution was activated for 20 s at the device’s highest intensity.

After the irrigation activation, each root specimen was flushed with 20 mL of sterile saline and gently dried with paper points. Subsequently, the samples were carefully extracted from their molds and placed into sterile Eppendorf tubes containing 1.5 mL of phosphate-buffered saline (PBS). They were then incubated at 37 °C for up to 7 days to facilitate TGF-β1 release. All experimental procedures were performed by a single endodontist who remained blinded to the specific irrigation solution applied.

### Measurement of TGF-β1 levels

Samples (100 µL from each Eppendorf tube) were collected from the incubated teeth on days 1 and 7 to quantify TGF-β1 levels and stored at –80 °C until analysis. TGF-β1 concentrations were measured using an enzyme-linked immunosorbent assay (ELISA, ELK Biotenchnology Company, Wuhan, China; Ref: ELK1185; sensitivity: 5.8 pg/mL; detection range: 15.63–1000 pg/mL), following the manufacturer’s instructions. Briefly, 100 µL of each thawed sample was added to the ELISA plate wells and incubated for 90 min at room temperature. After washing, the substrate and stop solution were applied. Absorbance was read at 450 ± 2 nm using a BIO-TEK ELx800 microplate reader (Vermont, USA), with washing performed by a BIO-TEK ELx50 washer. TGF-β1 levels were calculated from a standard curve. To ensure objectivity and minimize bias, all analyses were performed in a single-blinded manner.

### Scanning electron microscopy (SEM) analysis

To evaluate the effects of irrigation solutions and activation methods on dentin tubules, SEM analysis was performed. A total of nine teeth—one from each irrigation and activation group—were sectioned longitudinally. To prevent tubule contamination, teeth were split using a mouth spatula placed in grooves prepared on the buccal and lingual surfaces with a diamond disc. Samples were vacuum-dried and gold-coated using a sputter coater (SBC-800). SEM imaging was conducted at × 2500 magnification (HITACHI SU5000, Japan), focusing on the coronal third, which is associated with higher growth factor release [23].

### Statistical analysis

All statistical analyses were performed using SPSS 29.0 (SPSS Inc., Chicago, IL, USA). Normality and homogeneity were assessed with the Kolmogorov–Smirnov and Levene tests, respectively. As the data were not normally distributed, intergroup comparisons were made using the Kruskal–Wallis and Mann–Whitney U tests. The Wilcoxon rank-sum test was used for repeated measurements within groups. Percent changes in TGF-β1 levels were analyzed using one-way ANOVA and Kruskal–Wallis tests, with Tukey’s post-hoc and Mann–Whitney U tests for pairwise comparisons. A significance level of *p* < 0.05 was set.

## Results

**Fig. 1:**
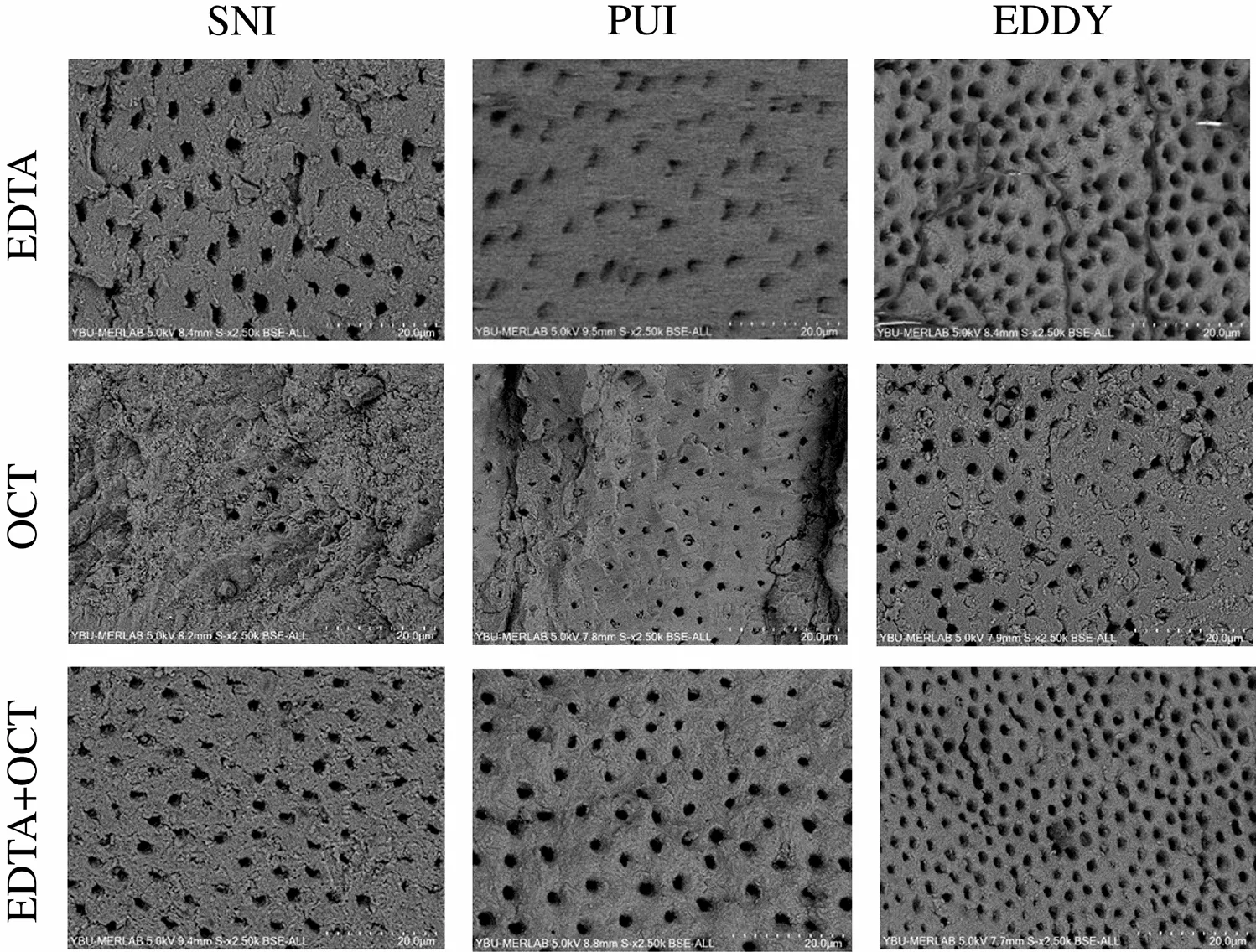
SEM images of dentin surfaces at × 2 500 magnification for each experimental group

SEM analysis demonstrated that irrigation with 17% EDTA, especially when activated by PUI or EDDY, effectively removed the smear layer and exposed patent dentinal tubules. In groups irrigated with 0.1% OCT, SNI showed greater smear layer retention, whereas PUI and EDDY activation enhanced smear layer removal and dentinal tubule openness comparable to EDTA. The use of EDTA followed by OCT resulted in enhanced smear layer elimination and increased dentinal tubule patency (Fig. 1).

Table 1 summarizes the TGF-β1 levels measured on days 1 and 7 for all irrigation solutions and activation methods. In all groups, TGF-β1 release significantly increased at day 7 compared to day 1 (*P* < 0.05). In the SNI group, 0.1% OCT resulted in significantly lower TGF-β1 release on day 1 compared to 17% EDTA and the 17% EDTA followed by 0.1% OCT group (*p* < 0.05). However, by day 7, the 17% EDTA followed by 0.1% OCT group showed the highest TGF-β1 levels among all solution groups (*p* < 0.05). In the PUI group, 0.1% OCT led to significantly lower release than the 17% EDTA followed by 0.1% OCT group on day 1 (*p* < 0.05), while 17% EDTA and the 17% EDTA followed by 0.1% OCT showed comparable levels (*p* > 0.05). At day 7, the 17% EDTA followed by 0.1% OCT group again demonstrated significantly greater TGF-β1 release than either solution alone (*p* < 0.05). Similarly, in the EDDY group, the 17%EDTA followed by 0.1% OCT group consistently resulted in significantly higher TGF-β1 release than the individual solutions at both time points (*p* < 0.05), whereas 17% EDTA and 0.1% OCT alone produced similar levels at day 7 (*p* > 0.05).

**Table 1:** Mean and standard deviations of the TGF-β1 levels (pg/mL) of the experimental groups based on different sampling times

		EDTA	OCT	EDTA followed by OCT
SNI	Day 1	17.96 ± 2.97^1,a^	15.77 ± 1.23^2,a^	19.38 ± 2.61^1,a^
	Day 7	23.33 ± 2.35^1,b^	19.21 ± 3.53^1,b^	26.69 ± 4.11^2,b^
PUI	Day 1	18.65 ± 5.40^1,2,a^	16.48 ± 3.34^1,a^	19.71 ± 3.51^2,a^
	Day 7	24.56 ± 4.67^1,b^	20.31 ± 1.13^1,b^	30.96 ± 5.97^2,b^
EDDY	Day 1	19.39 ± 13.57^1,a^	16.86 ± 3.38^1,a^	21.65 ± 4.10^2,a^
	Day 7	26.23 ± 14.88^1,b^	22.95 ± 6.68^1,b^	41.30 ± 3.91^2,b^

Different superscript numbers in the same row indicate a significant difference among irrigation solutions (*p* < 0.05)

Different superscript letters in the same column indicate a significant difference among activation methods in different time intervals (*p* < 0.05)

Table 2 presents the percentage change in TGF-β1 levels from day 1 to day 7. In the SNI group, all solutions showed an increase over time, but no statistically significant differences were observed between them (*p* > 0.05). In the PUI group, 0.1% OCT alone resulted in a significantly lower percentage increase compared to the 17% EDTA followed by 0.1% OCT group (*p* < 0.05), while 17% EDTA showed a similar increase to both the 0.1% OCT and the 17%EDTA followed by 0.1% OCT groups (*p* > 0.05). In the EDDY group, the 17% EDTA followed by 0.1% OCT led to a significantly higher percentage increase in TGF-β1 release than either 17% EDTA or 0.1% OCT alone (*p* < 0.05), which themselves showed comparable increases (*p* > 0.05). Among activation methods, differences were only significant when the 17%EDTA followed by 0.1% OCT was used, with EDDY producing the highest increase in TGF-β1 release (*p* < 0.05).

**Table 2:** Mean percentage changes (%) of the TGF- β1 values of the experimental groups from Day 1 to Day 7

	EDTA	OCT	EDTA followed by OCT
SNI	31.78 ± 14.32^1^	29.92 ± 21.15^1^	38.36 ± 17.33^1,a^
PUI	35.65 ± 20.52^1,2^	26.80 ± 22.17^2^	46.77 ± 32.40^1,3,a^
EDDY	42.85 ± 21.76^1^	34.27 ± 14.19^1^	95.16 ± 29.80^2,b^

Different superscript numbers in the same row indicate a significant difference among irrigation solutions (*p* < 0.05)

Different superscript letters in the same column indicate a significant difference among activation methods (*p* < 0.05)

## Discussion

Irrigation solutions and activation methods used in RETs play a critical role in creating a biologically favorable environment necessary for successful regeneration [24]. Accordingly, various irrigation solutions and activation techniques have been explored in the literature, focusing on their impact on the release of bioactive molecules. In line with this, the present study aimed to evaluate the effects of EDTA and OCT—both individually and in sequential use—on the release of TGF-β1 from dentin when activated using PUI and EDDY. Based on the results, the null hypothesis was rejected, as the different irrigation protocols led to significantly variable levels of TGF-β1 release.

In the evaluation of GF release from dentin matrix, various models such as dentin powder, discs, and root segments have been employed in the literature [9, 25, 26]. In this study, root segments derived from single-rooted teeth with their external surfaces sealed have been favored for their closer simulation of clinical conditions, as they restrict GF release to the root canal walls directly exposed to irrigation and activation protocols [27]. Moreover, segments were uniformly enlarged up to an apical size of #100 to mimic an immature tooth model, minimize variability in dentin volume, and ensure consistent irrigant contact [20]. To minimize microbial and environmental variability and to isolate the effects of the irrigation solutions and activation methods on TGF-β1 release, all specimens were sterilized by autoclaving, thereby ensuring standardized experimental conditions across groups. This approach was adopted to minimize the influence of external and confounding factors, allowing the release of GFs to be primarily attributed to the irrigating solutions used and the activation protocols applied. Moreover, intracanal medicaments were not included in the present study, as previous evidence indicated that these agents can independently induce GF release from dentin, thereby acting as a confounding factor [28]. Finally, SEM analysis provided a clear visualization of tubule openness, revealing that groups with more open dentin tubules exhibited increased TGF-β1 release. This finding highlights the critical role of smear layer removal in facilitating GF release and reinforces the reliability of the biochemical data obtained in this study.

Although the effect of GF release of EDTA has been investigated previously [9, 20], the role of OCT remains unexplored, and this jeopardizes the comparisons of the results directly. In this study, regardless of activation method, 0.1% OCT resulted in significantly lower TGF-β1 release on day 1 compared to 17% EDTA and the 17%EDTA followed by 0.1% OCT solutions. This is likely due to OCT’s weaker demineralization capacity compared to the strong calcium-chelating effect of EDTA [29]. However, percentage changes did not differ between groups. This can be explained by the individual differences in dentin volume for each tooth [26]. The exact volume of the initial dentin reservoir was not precisely known, which may have directly influenced the amounts of TGF-β1 released [30]. Furthermore, the elevated TGF-β1 levels observed following application of the combined solution may be attributed to a synergistic interaction between the strong calcium-chelating action of EDTA and the mild acidic demineralization induced by OCT, collectively enhancing GF release [17]. Notably, the neutral-to-basic pH of EDTA is suboptimal for the activation of latent TGF-β1 sequestered within the dentin matrix [27]. Consequently, the adjunctive use of OCT, with its slightly acidic pH, may have facilitated the conversion of latent TGF-β1 to its active form, thereby potentiating GF availability [31].

In line with the other studies [19, 21], the activation of the solutions increased the GF release in this study. When OCT was activated using PUI and EDDY, TGF-β1 release reached levels comparable to those observed with EDTA alone. This suggests that the enhanced irrigant penetration and acoustic streaming effects associated with activation systems can compensate for OCT’s limited calcium-chelating ability, facilitating effective disruption of the smear layer and demineralization of dentin to promote the release of dentin-derived GFs, similarly to EDTA. Furthermore, activation of the 17% EDTA followed by 0.1% OCT solutions using the EDDY produced a significantly greater release of TGF-β1 compared to PUI, corroborating recent evidence demonstrating the superior efficacy of EDDY [32]. This outcome may be attributed to the superior cavitation and acoustic streaming effects produced by the EDDY, which generate an optimal hydrodynamic environment within the root canal system. However, stem cell survival and attachment represent another critical determinant for the success of RETs. Despite their importance, limited information is currently available regarding the potential influence of different irrigation techniques on stem cell behavior. It has been reported that effective removal of the smear layer could enhance stem cell attachment and viability, as the smear layer contains toxic residues of necrotic tissue and bacterial remnants [33]. Accordingly, the superior smear layer removal efficacy demonstrated by the irrigation activation systems in the present study may contribute to improved cellular responses, potentially in conjunction with the observed increase in GF release.

Since GF release typically occurs after the induction of apical bleeding rather than during irrigation [26], extended observation periods are necessary to measure the release levels. In this sense, TGF-β1 levels were measured at two time points, days 1 and 7. Accordingly, all groups demonstrated a significant increase in release over time, which is consistent with the findings of previous studies [21, 28]. This may highlight the temporal dynamics in GF release.

Although this study offers valuable insights regarding the effect of OCT, which has been recently suggested as an alternative irrigation solution in RETs [18], on TGF-β1 release under controlled experimental conditions, several factors should be taken into account when interpreting the results. First, the complex biological relationship between pulpal and periradicular conditions and the presence, availability, and biological activity of dentin-derived growth factors cannot be fully replicated in an in vitro model. Clinical factors such as inflammation, infection, age, tooth type, or previous physiological conditions may influence both the baseline concentration and the release dynamics of GF within dentin. Moreover, since the exact quantity of GF released from dentin cannot be directly quantified, the measurement of GF release through its diffusion into PBS was employed as an indirect evaluation method. In addition, although OCT ensures a biocompatible environment in RETs, its possible effects on blood clotting and stem cells are still unclear. Finally, although teeth were prepared to the same size, minor anatomical variations and inconsistencies in internal root canal volume could have impacted the results, leading to differences in the amount of GF released between samples.

Based on the present findings of the study, future studies should focus on the effects of sequential use of EDTA and OCT-based irrigation protocols in more biologically complex models incorporating biofilm formation, intracanal medicaments, and blood clot or scaffold components to better simulate clinical RET conditions. In addition, evaluating the influence of these irrigation and activation strategies on stem cell viability, attachment, and differentiation would provide further insight into their biological relevance beyond GF release alone. Finally, well-designed clinical studies are needed to validate the translational potential of the present findings and to clarify the long-term regenerative outcomes associated with OCT-containing irrigation protocols in RETs.

## Conclusion

The sequential use of 17% EDTA and 0.1% OCT significantly enhanced TGF-β1 release compared to either solution alone, with EDDY activation further amplifying this effect. Given its limited chelating capacity, 0.1% OCT may serve as a complementary irrigant in RETs. Further clinical studies are warranted to validate these findings and assess their translational relevance in clinical practice.

## Data Availability

The data that support the findings of this study are available from the corresponding author upon reasonable request.
